# The Susceptibility of Retinal Ganglion Cells to Glutamatergic Excitotoxicity Is Type-Specific

**DOI:** 10.3389/fnins.2019.00219

**Published:** 2019-03-15

**Authors:** Ian Christensen, Bo Lu, Ning Yang, Kevin Huang, Ping Wang, Ning Tian

**Affiliations:** ^1^VA Salt Lake City Health Care System, Salt Lake City, UT, United States; ^2^Department of Ophthalmology & Visual Sciences, University of Utah School of Medicine, Salt Lake City, UT, United States

**Keywords:** glutamate excitotoxicity, retinal ganglion cell, susceptibility, cell type specific death, retinal diseases

## Abstract

Retinal ganglion cells (RGCs) are the only output neurons that conduct visual signals from the eyes to the brain. RGC degeneration occurs in many retinal diseases leading to blindness and increasing evidence suggests that RGCs are susceptible to various injuries in a type-specific manner. Glutamate excitotoxicity is the pathological process by which neurons are damaged and killed by excessive stimulation of glutamate receptors and it plays a central role in the death of neurons in many CNS and retinal diseases. The purpose of this study is to characterize the susceptibility of genetically identified RGC types to the excitotoxicity induced by *N*-methyl-D-aspartate (NMDA). We show that the susceptibility of different types of RGCs to NMDA excitotoxicity varies significantly, in which the αRGCs are the most resistant type of RGCs to NMDA excitotoxicity while the J-RGCs are the most sensitive cells to NMDA excitotoxicity. These results strongly suggest that the differences in the genetic background of RGC types might provide valuable insights for understanding the selective susceptibility of RGCs to pathological insults and the development of a strategy to protect RGCs from death in disease conditions. In addition, our results show that RGCs lose dendrites before death and the sequence of the morphological and molecular events during RGC death suggests that the initial insult of NMDA excitotoxicity might set off a cascade of events independent of the primary insults. However, the kinetics of dendritic retraction in RGCs does not directly correlate to the susceptibility of type-specific RGC death.

## Introduction

In mammals, retinal ganglion cells (RGCs) are the only output neurons that conduct visual signals from the eyes to the brain and they are classified into at least 40 types using a combination of morphological, functional and genetic features (Badea and Nathans, 2004; Völgyi et al., 2005; Kim et al., 2008; Briggman and Euler, 2011; Briggman et al., 2011; Kay et al., 2011; Sanes and Masland, 2015; Baden et al., 2016; Rheaume et al., 2018). RGC degeneration occurs in many retinal diseases leading to blindness. Increasing evidence suggests that RGCs are susceptible to various injuries in a type-specific manner. For instance, in experimental models of ocular hypertension, OFF RGCs exhibited higher rates of cell death compared to ON RGCs (Della Santina et al., 2013; El-Danaf and Huberman, 2015; Ou et al., 2016), and mono-laminated ON RGCs were found to be more susceptible to elevated IOP than bi-laminated ON-OFF cells (Feng et al., 2013). Similarly, in models of optic nerve injury, OFF RGCs were found to be more susceptible than ON RGCs, and ON sustained RGCs seem to be more susceptible than ON transient RGCs (Puyang et al., 2017). In addition, αRGCs seem to be the least susceptible RGC type to optic nerve injury in one report (Duan et al., 2015) but more susceptible RGC type in another report (Daniel et al., 2018). Recent studies have suggested that different types of RGCs could have unique gene expression patterns (Siegert et al., 2009; Madisen et al., 2012; Sanes and Masland, 2015) and the same genes expressed by RGCs could protect some type of RGCs but facilitate the death of other types of RGCs (Norsworthy et al., 2017). Thus, an understanding of the type-specific vulnerability of RGCs based on their gene expression may provide insights into the molecular mechanisms of neurodegeneration and suggest novel treatment strategies.

Glutamate excitotoxicity is the pathological process by which neurons are damaged and killed by excessive stimulation of glutamate receptors, such as the *N*-methyl-D-aspartate (NMDA) receptor, and it plays a central role in the death of neurons in many CNS diseases (Camacho and Massieu, 2006; Hulsebosch et al., 2009). Excessive stimulation of NMDA receptors can cause excitotoxicity by allowing high levels of calcium ions (Ca^2+^) to enter into cells (Manev et al., 1989). Ca^2+^ influx into cells activates a number of enzymes, including phospholipases, endonucleases, and proteases. These enzymes can damage cell structures such as the cytoskeleton, cell membrane, and DNA (Stavrovskaya and Kristal, 2005; Dutta and Trapp, 2011). In addition, a calcium influx through NMDA receptors can cause apoptosis through activation of a cAMP response element binding (CREB) protein shut-off (Hardingham et al., 2002). In the retina, NMDARs are expressed by all RGCs (Fletcher et al., 2000; Zhang and Diamond, 2009) and NMDA excitotoxicity is thought to cause RGC death in several retinal diseases (Kuehn et al., 2005; Kwon et al., 2009; Almasieh et al., 2012; Tezel, 2013; Evangelho et al., 2017). However, to what extent NMDA excitotoxicity causes the death of various types of RGCs has not been systematically investigated. Accordingly, we characterized the type-specific susceptibility of RGCs to NMDA excitotoxicity using several transgenic mouse lines, which express green/yellow fluorescent protein (GFP/YFP) in specific types of RGCs.

Our results show that the susceptibility of RGCs to NMDA excitotoxicity varies significantly among different types of RGCs. Among the RGCs studied, the J-RGCs have the highest susceptibility and the αRGCs have the lowest susceptibility. These results provide for the first time a direct comparison of the susceptibility of genetically identified types of RGCs to NMDA excitotoxicity.

## Materials and Methods

### Animals

The transgenic mouse strains used in this study include B6.Cg-Tg(Thy1-YFP)HJrs/J (Thy1-YFP), Tg(Thy1-EGFP)MJrs/J (Thy1-GFP), B6.129(SJL)-Kcng4^tm1.1(cre)Jrs^/J (Kcng4^Cre^), FSTL4-CreER (BD-CreER), JamB-CreER, TYW3, Thy1-STOP-loxP-YFP (Thy1-Stop-YFP). The Thy1-YFP, Thy1-GFP, and Kcng4^Cre^ mice were obtained from The Jackson Laboratory (Bar Harbor, ME, United States) (Duan et al., 2015). BD-CreER, JamB-CreER, TYW3, and Thy1-Stop-YFP mice were obtained from Dr. Joshua Sanes’ laboratory at Harvard University (Kim et al., 2008, 2010). All transgenic mice used in this study were on C57BL/6 background and were backcrossed with C57BL/6J mice for 4–5 generations in our lab. Then the BD-CreER, JamB-CreER and Kcng4^Cre^ mice were bred into the Thy1-Stop-YFP mice to generate BD-CreER:Thy1-Stop-YFP (BD:YFP), JamB-CreER:Thy1-Stop-YFP (JamB:YFP) and Kcng4^Cre^:Thy1-Stop-YFP (Kcng4^Cre^:YFP) double transgenic mice. YFP was expressed specifically in αRGCs without any additional treatment whereas YFP was only expressed specifically in BD-RGCs or J-RGCs upon intraperitoneal (IP) injection of Tamoxifen (150 μg) at the ages of P5-15. All of these mice were viable and no significant defects in general development or overall formation of eye or retina were noticed. All animal procedures used in this study and care were preformed following protocols approved by the IACUC of the University of Utah and the IACUC of VA Salt Lake City Health Care System in compliance with PHS guidelines and with those prescribed by the Association for Research in Vision and Ophthalmology (ARVO).

### Intraocular Injection of NMDA

The glutamate receptor agonist, NMDA, was injected intraocularly into the mice to induce *in vivo* glutamate excitotoxicity. The procedure of intraocular injection has been described previously (Xu et al., 2010). The actual dosage of the injected NMDA varied in concentration from 0.375 to 6.25 mmol/L but with a constant volume of 2 μl solution, which equivalent to 0.75–12.5 nmol of NMDA molecules. These amounts of NMDA injected into each eye are similar to those used in previous studies (Bai et al., 2013; Kimura et al., 2015; Jiang et al., 2016; Zhao et al., 2016; Ishimaru et al., 2017; Wang et al., 2018). The distribution of the solution inside the eyes was confirmed by co-injecting NMDA with Alexa Fluor^TM^ 555 conjugated Cholera Toxin Subunit B (CTB, 0.2%, Thermo Fisher Scientific, Eugene, OR, United States) and the retinas were examined by imaging the distribution of the fluorescent signaling (data not shown). To reduce the impact of the variation of YFP expression in some of the transgenic mouse lines, we injected 2 μl NMDA solution into one eye (left) and used the non-injected contralateral eyes (right) as controls to calibrate the cellular survival rate of each mouse. In preparation for intraocular injection, the mice were anesthetized with Isoflurane (1–5% Isoflurane mixed with room air delivered in a rate between 0.8 and 0.9 L/min) through a mouse gas anesthesia head holder (David KOPF Instruments, Tujunga, CA, United States) and local application of 0.5% proparacaine hydrochloride ophthalmic solution on each eye. Glass micropipettes made from borosilicate glass using a Brown-Flaming horizontal puller with fine tip (about 10–15 μM diameter) were used for injection. The glass needle was mounted on a Nano-injection system (Nanoject II, Drummond Scientific Company, Broomall, PA, United States), which could precisely control the amount of injected solution at the nl level. The glass needle was aimed to penetrate the eyeball near its equator under a dissection microscope and a total of 2 μl solution was slowly injected into each eye. After the injection, the eyes were covered with 0.5% erythromycin ophthalmic ointment and the mice were placed in a clean cage siting on a water blanket. The temperature of the water blanket was set at 33°C. Mice in this cage were continuously monitored until they completely recovered and then they were returned to their original cages. The procedures for anesthesia and intraocular injection fit the procedures approved by the IACUC of the University of Utah and the IACUC of VA Salt Lake City Health Care System.

### Primary Antibodies

Rabbit polyclonal antibody against green fluorescent protein (GFP) conjugated with AlexaFluor 488 was purchased from Molecular Probes (Eugene, OR, United States; Catalog No. A21311). This antibody was raised against GFP isolated directly from *Aequorea victoria* and has been previously characterized by immunocytochemistry in granule cells (Overstreet-Wadiche et al., 2006), olfactory sensory neurons (Lèvai and Strotmann, 2003), and hippocampal neurons that express GFP (Huang et al., 2005). Anti-active Caspase-3 antibody (anti-CASP3) was purchased from Abcam (Cambridge, MA, United States; Catalog No. ab2302). This polyclonal antibody was raised in rabbits against synthetic peptide corresponding to the N-terminus adjacent to the cleavage site of human active caspase-3 preferentially recognizes the p17 fragment of the active Caspase-3 and has been characterized by immunocytochemistry and Western blotting. Anti-RBPMS (RNA binding protein with multiple splicing) antibody was purchased from PhosphoSolutions (Aurora, CO, United States; Catalog #: 1832-RBPMS). This polyclonal antibody was raised in guinea pigs against synthetic peptide corresponding to amino acid residues from the N-terminal region of the rat RBPMS sequence conjugated to KLH. This antibody has been characterized by Western blotting and verified with immunocytochemistry on mammalian retinas (Kwong et al., 2010; Rodriguez et al., 2014). The secondary antibodies were purchased from Jackson Immune Research Laboratories (West Grove, PA, United States).

### Preparation of Retinal Whole-Mounts for Antibody Staining

Retinal ganglion cells were imaged on whole mount retinal preparation for cell counting and dendritic morphology. The procedures for fluorescent immuno-labeling of YFP-expressing retinal neurons on retinal whole-mounts and slide preparations have been described previously in detail (Xu et al., 2010). In brief, mice were euthanized with 100% CO2 followed by cervical dislocation. Retinas were isolated and fixed in 4% paraformaldehyde (PFA) in 0.01M phosphate-buffered saline (PBS; pH 7.4) for 30 min at room temperature. Fixed retinas were washed 10 min × 3 in 0.01M PBS and incubated in blocking solution (10% normal donkey serum) at 4°C for 2 h. Next, retinas were incubated in a rabbit polyclonal anti-GFP antibody conjugated with Alexa Fluor488 (1:500) for 7 days at 4°C.

In one experiment, the total RGCs were labeled by a guinea pig polyclonal anti-RBPMS antibody (1:500) and the YFP-expressing RGCs were labeled by a rabbit anti-GFP antibody conjugated with AlexaFluor 488. A Cyanine Cy^TM^ 3-conjugated donkey anti-guinea pig (1:400, Jackson ImmunoResearch, West Grove, PA, United States) secondary antibody was used overnight at 4°C to reveal anti-RBPMS antibody staining. In another experiment, a rabbit polyclonal anti-Caspase 3 antibody (1:150) was used to label YFP/GFP expressing RGCs actively undergoing apoptosis. An Alexa 647-conjugated donkey anti-rabbit (1:300, Jackson ImmunoResearch, West Grove, PA, United States) secondary antibody was used overnight at 4°C to reveal anti-Caspase 3 antibody staining. In this experiment, YFP/GFP signals in RGCs were not enhanced by anti-GFP antibody but the YFP/GFP expressing RGCs were still identifiable with confocal imaging. After the antibody incubation, the retinas were washed 3 × 10 min, and flat mounted on Super-Frost slides (Fisher Scientific, Pittsburgh, PA, United States) with Vectashield mounting medium for fluorescence (Vector Laboratories, Burlingame, CA, United States).

### Confocal Laser Scanning Microscopy and Image Sampling

Fluorescent images of fixed retinal tissue were collected with a dual-channel Zeiss confocal microscope (Carl Zeiss AG, Germany) with the C-Apochromat 40× 1.2WKorr water immersion lens. Image stacks of YFP expressing RGCs in whole-mount retinas were collected at intervals of 0.5 μm. Imaris software (Bitplane, Inc., Concord, MA, United States) was used to align multi-stacks of images together, quantify the number and dendritic structure of RGCs, and adjust the intensity and contrast of images.

**FIGURE 1:**
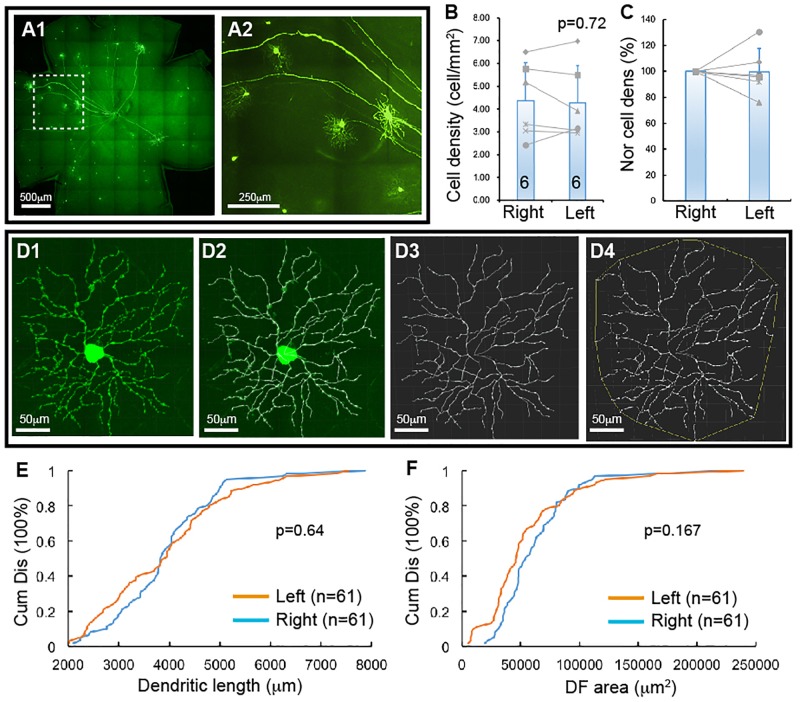
YFP-expressing RGCs of Thy1-YFP mice. The density and dendritic structure of YFP-expressing RGCs of Thy1-YFP mice were quantified and compared between left and right eyes. **(A)** Representative retina image of a Thy1-YFP mouse **(A1)** and a magnified view of the area in the dash-line box of **(A1)** to show the morphology of YFP-positive RGCs **(A2)**. **(B)** Comparison of the average densities of YFP-expressing RGCs of left and right eyes of the same mice [paired *t*-test, *p* = 0.72, number of mice for each group (*n*) = 6]. The gray lines indicate six pairs of RGC density of left and right eyes. **(C)** Normalized RGC density of the same six mice shown in **(B)**. The RGC density of the left eye of each mouse is normalized to the right eye of the same mouse and the RGC density of right eyes is “self-normalized.” The gray lines indicate six pairs of normalized RGC densities. **(D)** A representative image of a YFP-expressing RGC **(D1)**, the overlay of the dendrites and the tracing results **(D2)**, the tracing result **(D3)**, and the measurement of the dendritic field (DF) **(D4)**. **(E)** Paired comparison (K–S test, *p* = 0.64) of the cumulative distribution of the dendritic length of YFP-expressing RGCs of left and right eyes of the same six mice shown in **(B)**. **(F)** Paired comparison (K–S test, *p* = 0.167) of the cumulative distribution of the size of DF of YFP-expressing RGCs of the left and right eyes of the same six mice. n, the number of cells for each group in **(E,F)**.

For image sampling, we use two different strategies for retinas with low or high densities of YFP-expressing RGCs to avoid potential bias of data sampling when the persons carrying out the histological analysis were not blinded to the treatment. For Thy1-YFP, BD:YFP and JamB:YFP mice, the YFP is expressed in a relatively low density of RGCs and the expression level varies significantly among mice (from several to several hundreds of YFP-expressing RGCs per retina) but not significantly between left and right eyes (see results in Figure 1B, 3B). Therefore, we imaged the whole retina and counted every YFP-expression RGCs in the GCL layer of these mice. The only case to exclude a mouse from data analysis is when the total number of YFP-expressing RGCs in the whole retina of the control eye is less than 10 to avoid the results to be skewed by mice with extreme low number of YFP-expressing RGCs. For TYW3 and Kcng4^Cre^:YFP mice, which constitutively express YFP in all W3-RGCs and αRGCs, the density of YFP+ RGCs is very high (several thousands per retina) and the expression level does not very significantly among mice or between left and right eyes (see results in Figure 3F, 4A). We included every mouse assigned to this study for data analysis without exclusion. For image sampling, we scanned 4 squares (304 μm × 304 μm each) at 4 quarters of the retina 600 μm away from the center of optic nerve head (see Figure 3E for details). The density of YFP-expressing W3-RGCs and αRGCs of each retina was averaged from the 4 squares.

### Preparation of Retinal Whole-Mounts for *ex vivo* Fluorescent Imaging

The time-lapse images of RGCs were taken from whole-mount retina preparations as previously described (Xu et al., 2010). Retinas of Thy1-YFP mice, BD:YFP mice and JamB:YFP mice were used for *ex vivo* imaging of αRGCs, BD-RGCs and J-RGCs, respectively. Retinas were isolated from Thy1-YFP, BD:YFP and JamB:YFP mice in oxygenated extracellular solution that contained (in mmol/L) NaCl 124, KCl 2.5, CaCl2 2, MgCl2 2, NaH2PO4, 1.25, NaHCO3 26, and glucose 22 (pH 7.35 with 95% O2 and 5% CO2), mounted on nitrocellulose filter paper (Millipore Corp), placed in a recording chamber and continuously perfused at 32°C. Image stacks were taken using a two-photon image system (Prairie Technologies, Inc., Middleton, WI, United States) immediately before bath application of NMDA. After 10 min of bath application of 200 nmol/L NMDA, the retinas were continuously perfused with the oxygenated extracellular solution and the cells were imaged every 1 h for 7 h. The dendritic density of imaged cells is measured using a Sholl analysis (Xu et al., 2010) and the dendritic density after NMDA application was normalized to pre-NMDA application (0 h).

### Statistical Analysis

Data are all presented as mean ± SE in the text and figures. Student *t*-tests are used to examine the difference between two means, K–S test is used to examine the difference between two cumulative distributions.

## Results

### RGCs Lose Dendrites Before They Die Due to NMDA Excitotoxicity

Thy1-YFP mice have been used extensively for studying RGC morphology, physiology, development and degeneration. One potential advantage is that YFP is expressed in about 12 morphological types of RGCs in this mouse (Xu et al., 2010). To determine whether this mouse could serve as a model to study type specific RGC death in retinal diseases, we quantified the number and dendritic structure of YFP-expressing RGCs with the approach we previously used in our study (Xu et al., 2010). Figure 1A shows a representative image of a flat-mount retina of a Thy1-YFP mouse and a magnified area of the retina. The number and dendritic structure of the YFP-expressing RGCs are readily quantifiable using confocal imaging. Figure 1B shows the densities of YFP-expressing RGCs of left and right eyes of 6 Thy1-YFP mice. Although a paired t-test showed that the difference between the densities of YFP-expressing RGCs of left and right eyes is statistically insignificant (paired *t*-test, *p* = 0.72), it seems that the density of YFP-expressing RGCs varies significantly among these mice. To determine the variation of the densities of YFP-expressing RGCs of the left and right eyes of the same mouse, we normalized the density of YFP-expressing RGCs of the left eye to that of the right eye of the same mouse. Figure 1C shows the normalized densities of YFP-expressing RGCs of left and right eyes of 6 Thy1-YFP mice. We then quantified the dendritic length and the size of dendritic field (DF) of YFP-expressing RGCs (Figure 1D) and found that the distributions of dendritic length and the size of DF of left and right eyes are not statistically different (Figure 1E,F, K–S tests, *p* = 0.64 and 0.167, respectively). Therefore, the number and dendritic properties of YFP-expressing RGCs of the left and right eyes are comparable.

**FIGURE 2:**
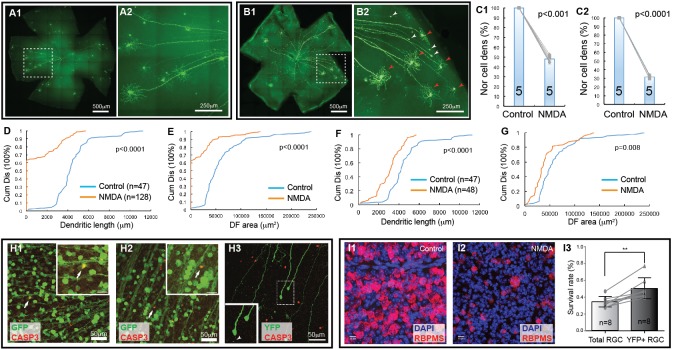
Retinal ganglion cell death in NMDA excitotoxicity of mice. The density and dendritic structure of YFP-expressing RGCs of Thy1-YFP mice with and without NMDA injections were quantified and compared. **(A)** Representative image of a flat mount retina of a Thy1-YFP mouse without NMDA injections **(A1)** and a magnified view of the area in the dash-line box of panel A1 to show the morphology of YFP-positive RGCs **(A2)**. Red arrowheads indicate survival RGCs and white arrowheads indicate remaining axonal segment of RGCs without soma and dendrites. **(B)** Representative image of a flat mount retina of a Thy1-YFP mouse with NMDA injections **(B1)** and a magnified view of the area in the dash-line box of **(B1)** to show death of YFP-positive RGCs **(B2)**. Red arrowheads indicate survival RGCs and white arrowheads indicate remaining axonal segment of RGCs without soma and dendrites. **(C)** Comparison of the normalized density of YFP-expressing RGCs that survived 24 h after 2 μl of 3.125 mmol/L (6.25 nmol, **C1**, paired *t*-test, *p* < 0.001) or 6.25 mmol/L (12.5 nmol, **C2**, paired *t*-test, *p* < 0.0001) NMDA injection to the contralateral control eyes. The numbers in the columns indicate the number of eyes tested. The cell density of the left eye of each mouse is normalized to the right eye of the same mouse. The gray lines indicate six pairs of normalized RGC densities. **(D)** Comparison of the cumulative distributions of the dendritic length of all YFP-expressing RGCs (with and without identifiable dendrites) that survived 24 h after 6.25 mmol/L (12.5 nmol) NMDA injection (left eyes) with the non-injected right eyes of the same mice (5 mice; *n* = number of RGCs for each group) (K–S test, *p* < 0.0001). Because the non-injected eyes have higher density of YFP-expressing RGCs with intact dendritic tree and they overlap more frequently, the number of RGCs can be traced are fewer than that NMDA injected eyes. **(E)** Comparison of the cumulative distributions of the size of the DF of the same two groups of YFP-expressing RGCs as shown in **(D)** (K–S test, *p* < 0.0001). **(F)** Comparison of the cumulative distributions of the dendritic length of the YFP-expressing RGCs with identifiable dendrites after 6.25 mmol/L (12.5 nmol) NMDA injection with the non-injected right eyes of the same mice (the same 5 mice as for data presented in **(D)**; *n* = number of RGCs for each group, K–S test, *p* < 0.0001). **(G)** Comparison of the cumulative distributions of the size of the DF of the same two groups of YFP-expressing RGCs as shown in **(F)** (K–S test, *p* = 0.008). **(H)** **(H1,H2)** show magnified views of the retinas of Thy1-GFP mice, in which most RGCs are GFP-expressing, treated with NMDA *in vivo* for 3 **(H1)** and 6 **(H2)** hours. The retinas are labeled with anti-GFP (green) and anti-CASP3 (red) antibodies showing some GFP-positive cells are also CASP3-positive. H3 shows a magnified view of the retina of a Thy1-YFP mouse, in which only a small fraction of RGCs are YFP-expressing, treated with NMDA *in vivo* for 6 h and labeled with anti-GFP (green) and anti-CASP3 (red) antibodies. Many YFP-expressing RGCs with no dendrites are still CASP3-negative. Inserts show the enlargement of the cells indicated by the arrows/dash-line box. Insert of **H3** shows two RGCs and one of them completely lost all dendrites but another one has one short dendritic branch remaining (white arrowhead). **(I)** Magnified views of flat mount retinas of Thy1-YFP mice with **(I2)** and without **(I1)** 3.125 mmol/L (6.25 nmol) NMDA injection. The total RGCs were labeled by anti-RBPMS antibody and compared with YFP-expressing RGCs in the same retina (YFP labeling images not shown). Survival rates of total RGCs and YFP-expressing RGCs 24 h after NMDA injection were derived by normalizing the total RGCs and YFP-expressing RGCs of NMDA injected left eyes to the non-injected right eyes of the same mice and compared using a paired *t*-test (**I3**, *n*, number of mice, *p* = 0.002). ^∗∗^indicate 0.05 > p > 0.001.

Next, we tested whether the Thy1-YFP mice could be used to determine type-specific RGC death under disease conditions. Accordingly, we injected NMDA solution into the left eyes of Thy1-YFP mice and use the non-injected right eyes as controls, quantified the number of YFP-expressing RGCs and their dendritic properties, and compared the results of NMDA treated left eyes to that of non-injected right eyes. Figure 2A shows representative images of Thy1-YFP retinas without NMDA injection while Figure 2B shows a retina 1 day after NMDA injection. It is evident that many YFP-expressing RGCs died 1 day after NMDA injection and that a significant number of the surviving YFP-expressing RGCs lost dendrites (Figure 2B2,H3). Quantitatively, the densities of YFP-expressing RGCs are reduced to 48.1 ± 3.4% (paired *t*-text, *p* < 0.001) and 31.6 ± 2.1% (paired *t*-text, *p* < 0.0001) in retinas with intraocular injection of 2 μL 3.125 mmol/L (6.25 nmol) or 6.25 mmol/L (12.5 nmol) NMDA as compared to the non-injected right eyes (Figure 2C1,C2), respectively. More than 60% of the surviving YFP-expressing RGCs completely lost their dendrites with intraocular injection of 2 μL 6.25 mmol/L (12.5 nmol) NMDA (Figure 2D,E, K–S test, *p* < 0.0001). In addition, among the surviving YFP-expressing RGCs with dendrites, the total dendritic length and the size of DF are significantly reduced (Figure 2F,G, K–S test, *p* < 0.0001 or = 0.008, respectively). These results demonstrate that RGCs lose dendrites before death, which is consistent with previous reports that RGCs significantly lose or re-organize their dendrites under various disease conditions (Kuehn et al., 2005), such as glaucoma (Weber et al., 1998; Shou et al., 2003; Morgan et al., 2006; Williams et al., 2013) and optic nerve crush (ONC) (Leung et al., 2008; Weber et al., 2008).

To further test this idea, we injected NMDA into eyes of Thy1-GFP mice, in which most, if not all, RGCs are GFP-expressing, and labeled NMDA-treated retinas using an anti-CASP3 antibody to identify cells undergoing apoptosis (Pi et al., 2018). The results show that some GFP-positive cells are also CASP3-positive 3 h after NMDA injection (Figure 2H), indicating that RGCs actively undergoing apoptosis are still GFP positive, while no CASP3-positive RGCs are found in non-injected eyes (data not shown). We also labeled NMDA-treated retinas of Thy1-YFP mice, in which only a small fraction of RGCs are YFP-expressing, with the anti-CASP3 antibody and found that many YFP-expressing RGCs with no dendrites are still CASP3-negative (Figure 2H3). These results further demonstrate that the damaged RGCs lose all dendrites before the beginning of apoptosis. Because the YFP expressed by RGCs are cytosol protein and can fill the fine remnants of dendrites of the injured RGCs (Figure 2H3, white arrow in the insert) as well as soma and axon, the changes in YFP-positive dendrites are likely to reflect the changes of dendritic morphology of the RGCs but not the expression level of YFP in responding to NMDA excitotoxicity. Since RGCs lose dendrites prior to death, the Thy1-YFP mice appear to be an unreliable model for studying type-specific RGC death based on dendritic morphology.

Furthermore, to determine whether the death of YFP-expressing RGCs of Thy1-YFP mice could represent the death of total RGCs induced by NMDA excitotoxicity, we compared the survival rate of YFP-expressing RGCs and total RGCs labeled by anti-RBPMS antibody (Figure 2I1,I2) of Thy1-YFP mice treated by intraocular injection of 2 μl 3.125 mmol/L (6.25 nmol) NMDA into the left eyes with non-injected right eyes. Our results show that the survival rate of YFP-expressing RGCs (50%) is significantly higher than that of anti-RBPMS antibody labeled RGCs (34.2%) (Figure 2I3, paired *t*-test, *p* = 0.002), which is within the range of the results of several previous studies (Bai et al., 2013; Kimura et al., 2015; Jiang et al., 2016; Zhao et al., 2016; Ishimaru et al., 2017; Wang et al., 2018). These results suggest that the susceptibility of YFP-expressing RGCs in this mouse line is lower than the susceptibility of total RGCs. Therefore, Thy1-YFP mice seem to be an unreliable model for studying overall RGC death. Accordingly, we further employed four RGC type-specific transgenic mouse lines to study type-specific RGC death due to NMDA excitotoxicity.

### The Dose-Response Relationship of NMDA Excitotoxicity Induced RGC Death

**FIGURE 3:**
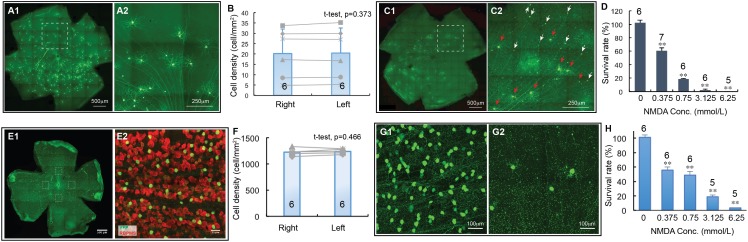
The dose-response relationship of BD-RGCs and W3-RGCs to NMDA excitotoxicity. The density of YFP-expressing RGCs in BD:YFP and TYW3 mice with NMDA injection into left eyes at various concentrations were quantified, normalized to the non-injected right eyes and presented as a function of the concentrations of NMDA injected. **(A)** A representative image of a BD:YFP mouse retina without NMDA injection **(A1)** and a magnified view of the dash-line box of **(A1)** to show the morphology of BD-RGCs **(A2)**. **(B)** Comparison of the density of YFP-expressing BD-RGCs of untreated right and left eyes [number of mice (n) = 6, paired *t*-test, *p* = 0.373]. **(C)** A representative image of a BD:YFP mouse retina 24 h after 2 μl 0.75 mmol/L (1.5 nmol) NMDA injection **(C1)** and a magnified view of the dash-line box of **(C1)** to show the survival BD-RGCs with dendrites (red arrows) and axonal remnants of dead RGCs (white arrows) **(C2)**. **(D)** The survival rates of BD-RGCs treated with four different concentrations of NMDA. The survival rate of each NMDA treated left eye was normalized to the non-injected right eye of the same mouse. The average survival rate of eyes treated by each concentration of NMDA was compared to the untreated contralateral eyes of the same group of mice using paired *t*-test (*n*, number of mice). **(E)** A representative image of a flat-mount TYW3 mouse retina without NMDA injection (**E1**, only showing YFP staining but not anti-RBPMS staining). For RGC counting, we imaged 4 squares (304 μm × 304 μm each) at 4 quarters of the retina 600 μm away from the center of optic nerve head (dash-line boxes). **(E2)** Shows a magnified view of the dash-line box on the right side of **(E1)** to show the anti-RBPMS staining of all RGCs (red) and YFP-expressing W3 RGCs (green). **(F)** Comparison of the density of YFP-expressing W3-RGCs of untreated left and right eyes of TYW3 mice [number of mice (*n*) = 6, paired *t*-test, *p* = 0.466]. **(G)** Magnified views of TYW3 mouse retinas with **(G2)** and without **(G1)** 6.25 mmol/L (12.5 nmol) NMDA injection. **(H)** The survival rates of W3-RGCs treated with four different concentrations of NMDA. The survival rate of each NMDA treated left eye was normalized to the non-injected right eye of the same mouse. The number in each column of **(B,F)** and the number on top of each column of **(D,H)** indicate the number of eyes of each group. In **(D,H)**, ^∗∗^indicates 0.05 > *p* > 0.001.

We first quantified cell death as a function of NMDA concentrations using two transgenic mouse lines, in which YFP is expressed by the BD-RGCs or W3-RGCs (Kim et al., 2010). BD:YFP mice express YFP in BD-RGCs and a small fraction of amacrine cells located in INL (Kim et al., 2010) but not displaced amacrine cells in the ganglion cell layer (GCL) (data not shown). In this study, we only included the YFP-expressing cells in the GCL. Figure 3A shows a representative image of a BD:YFP retina (A1) and a magnified area to show the dendritic morphology of the BD-RGCs (A2). Similar to Thy1-YFP mice, the density of YFP-expressing BD-RGCs varies significantly among mice. However, the densities of YFP-expressing BD-RGCs of the left and right eyes of the same mice are very similar (Figure 3B). A paired *t*-test showed that the difference between the densities of YFP-expressing BD-RGCs of left and right eyes of BD:YFP mice is statistically insignificant. Therefore, we treated the left eye with NMDA and compared the cell density with the non-injected right eye of each mouse.

BD-RGCs seem to be very sensitive to NMDA excitotoxicity. Figure 3C shows a representative image of a BD:YFP retina 24 h after intraocular injection of 2 μL 0.75 mmol/L (1.5 nmol) NMDA. Clearly, some BD-RGCs completely lose their dendrites and somas but retain axonal remnants 24 h after NMDA injection (C2, white arrows) while a significant fraction of BD-RGCs still retain their dendrites (red arrows). We quantified the survival rates of BD-RGCs to four different NMDA concentrations and found that the density of BD-RGCs was reduced to 39.5 ± 4.3% at 2 μL 0.375 mmol/L (0.75 nmol) to 0% at 2 μL 6.25 mmol/L (12.5 nmol) as compared to the non-injected right eyes (Figure 3D). The differences between the RGC densities in all four NMDA treated groups and their respective non-injected controls are statistically significant (paired *t*-test).

Similarly, we quantified the densities of W3-RGCs treated with the same four NMDA concentrations and compared with their respective non-injected controls. In TYW3 mice, YFP is expressed in the W3-RGCs constitutively with a very high density and, therefore, the number of YFP-expressing W3-RGCs does not vary significantly among mice or between the left and right eyes (Figure 3E,F, paired *t*-test, *p* = 0.466). Because YFP is also expressed in a very small fraction of amacrine cells located in the INL but not displaced amacrine cells in the GCL in these mice (data not shown), we only included the YFP-expressing cells in the GCL. W3-RGCs were also found to be very sensitive to NMDA excitotoxicity (Figure 3G). The density of W3-RGCs was reduced to 55.8 ± 4.3% at 0.375 mmol/L (0.75 nmol) and to 3.5 ± 0.1% at 6.25 mmol/L (12.5 nmol) NMDA in comparison to the non-injected right eyes (Figure 3H). The differences between the RGC densities in all four NMDA treated groups and their respective non-injected controls are statistically significant.

### αRGCs Are Relatively Resistant to NMDA Excitotoxicity

**FIGURE 4:**
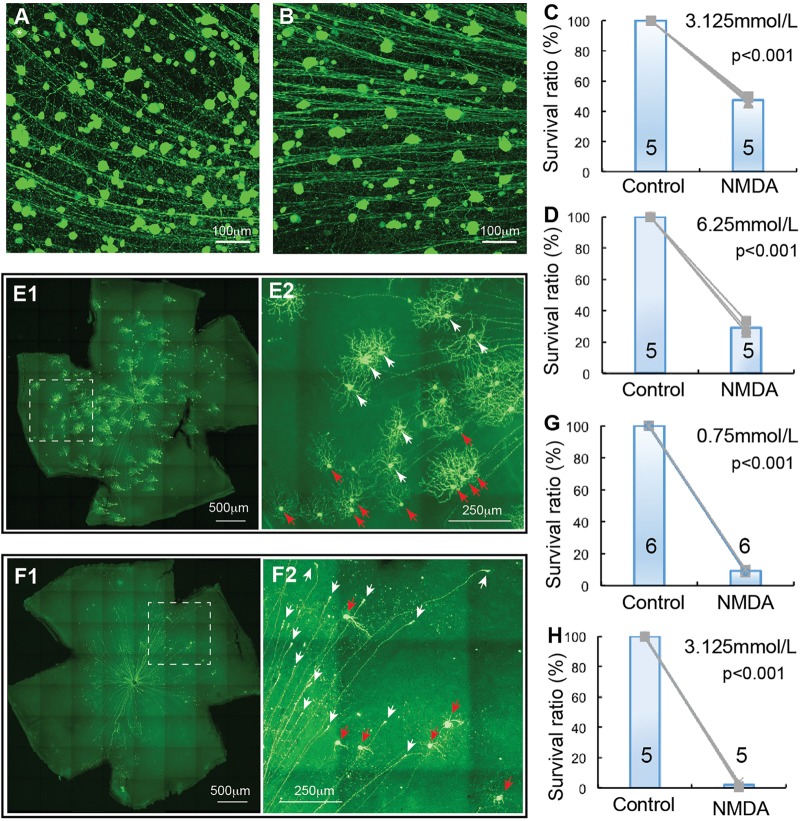
αRGC and J-RGC death in NMDA excitotoxicity. The density YFP-expressing αRGCs and J-RGCs with NMDA injection into left eyes were quantified and normalized to that of non-injected right eyes. **(A)** A magnified view of a Kcng4^Cre^:YFP mouse retina without NMDA injection to show YFP-expressing αRGCs. **(B)** A magnified view of a Kcng4^Cre^:YFP mouse retina 24 h after 2 μl 6.25 mmol/L (12.5 nmol) NMDA injection to show the death of αRGCs. **(C)** The normalized survival rates of αRGCs treated with 2 μl 3.125 mmol/L (6.26 nmol) NMDA injection and control eyes. **(D)** The survival rates of αRGCs treated with 6.25 mmol/L (12.5 nmol) NMDA injection and control eyes. **(E)** A representative image of a flat-mount JamB:YFP mouse retina without NMDA injection **(E1)** and a magnified view of the dash-line box of **(E1)** to show the morphology of J-RGCs **(E2)**, in which the J-RGCs with a more symmetric DF are indicated by white arrow-heads and the J-RGCs with an asymmetric DF are indicated by red arrow-heads. **(F)** A representative image of a flat-mount retina of a JamB:YFP mouse 24 h after 0.75 mmol/L (1.5 nmol) NMDA injection **(F1)** and a magnified view of the dash-line box of panel F1 to show the death of J-RGCs **(F2)**, in which survival J-RGCs with dendrites are indicated by red arrows and axonal remnants of dead RGCs are indicated by white arrows. **(G)** The normalized survival rates of J-RGCs treated with 2 μl 0.75 mmol/L (1.5 nmol) NMDA injection and control eyes. **(H)** The survival rates of J-RGCs treated with 2 μl 3.125 mmol/L (6.25 nmol) NMDA injection and control eyes. The numbers in each column of **(C,D,G,H)** indicate the number of eyes of each group.

αRGCs have been reported to be relatively resistant to ONC (Duan et al., 2015). We investigated whether they are also more resistant to NMDA excitotoxicity than BD-RGCs and W3-RGCs. Similar to TYW3 mice, the number of YFP-expressing αRGCs in Kcng4^Cre^:YFP mice is highly consistency both between mice and between the left and right eyes (Figure 4A). In addition to αRGCs, YFP is also expressed in some bipolar cells in Kcng4^Cre^:YFP mice (Duan et al., 2015). In our study, we only included the YFP-expressing cells in the GCL. Clearly, αRGCs seem to be more resistant to NMDA excitotoxicity than BD-RGCs and W3-RGCs. Many αRGCs were still visible from Kcng4^Cre^:YFP retina 1 day after 2 μL 6.25 mmol/L (12.5 nmol) NMDA injection (Figure 4B). Quantitatively, intraocular injection of 2 μL 3.125 mmol/L (6.25 nmol) or 6.25 mmol/L (12.5 nmol) NMDA reduced the density of αRGCs to 47.4 ± 1.8% and 29.1 ± 3.9% of the non-injected right eyes, respectively (Figure 4C,D). The differences between the NMDA injected eyes and the non-injected eyes are statistically significant in both NMDA concentrations (paired *t*-test, *p* < 0.001).

### J-RGCs Are Highly Sensitive to NMDA Excitotoxicity

Finally, we examined the susceptibility of J-RGC to NMDA excitotoxicity using JamB:YFP mice. JamB:YFP mice express YFP in two types of J-RGCs with distinctive dendritic morphology, one with an asymmetric DF and another with a more symmetric DF (Kim et al., 2008, 2010). In addition, YFP is also expressed in some amacrine cells located in the INL but not displaced amacrine cells in the GCL (data not shown). In this study, we only count the YFP-expressing cells in the GCL, which includes both types of J-RGCs, but not YFP-expressing cells in the INL. Figure 4E shows a representative image of a JamB:YFP retina (E1) and a magnified view to show the dendritic morphology of the two types J-RGCs. J-RGCs seem to be extremely sensitive to NMDA excitotoxicity. Figure 4F shows a representative image of a JamB:YFP retina 1 day after intraocular injection of 2 μL 0.75 mmol/L (1.5 nmol) NMDA. In this JamB:YFP retina, the vast majority of J-RGCs lost their dendrites and somas and only retained their axonal processes 1 day after NMDA injection (Figure 4F2, indicated by white arrows). Quantitatively, intraocular injection of 2 μL 0.75 mmol/L (1.5 nmol) or 3.125 mmol/L (6.25 nmol) NMDA reduced the densities of J-RGCs to 9.3 ± 0.7% and 2.1 ± 1.2% in comparison with the non-injected right eyes, respectively (Figure 4G,H). The differences between the NMDA injected eyes and the non-injected eyes are highly significant with these two NMDA concentrations (paired *t*-test, *p* < 0.001).

### The Susceptibility of RGCs to NMDA Excitotoxicity Is Type-Specific

**FIGURE 5:**
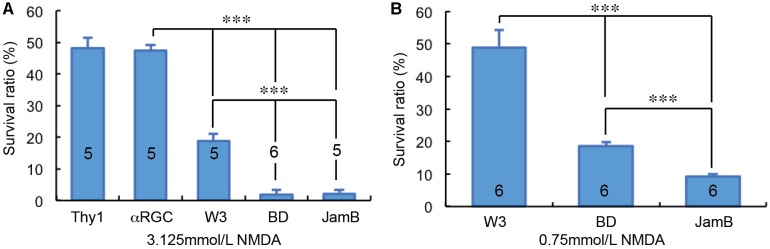
RGC type-specific susceptibility to NMDA excitotoxicity. The survival rates of YFP-expressing RGCs in Thy1-YFP mice, Kcng4^Cre^:YFP mice, TYW3 mice, BD:YFP and JamB:YFP mice 24 h after intraocular injection of 2 μl 3.125 mmol/L (6.25 nmol) NMDA **(A)** or 2 μl 0.75 mmol/L (1.5 nmol) NMDA **(B)** were compared to the contralateral control eyes. The number in each column indicates the number of eyes of the group. ^∗∗∗^ indicates 0.001 > *p*.

Overall, we examined the susceptibility of four groups of RGCs to NMDA excitotoxicity and the results showed a clear type-specific pattern of RGC death. Of the tested four groups of RGCs, αRGCs seem to be the most resistant RGCs to NMDA excitotoxicity, while J-RGCs are the most sensitive cells to NMDA excitotoxicity (Figure 5A,B). The survival rates of these four groups of RGCs varied from 47.4 ± 1.8% (αRGCs) to 9.3 ± 0.7% (J-RGCs) in response to 2 μL 3.125 mmol/L (6.25 nmol) NMDA. When the results are compared to the collective responses of 12 morphological types of RGCs from the Thy1-YFP mice, the survival rate of αRGCs is very close to that of 12 morphological types of YFP-positive RGCs of Thy1-YFP mice (Figure 5A). Paired tests show that the differences in RGC survival rates between the four groups of NMDA treated RGCs to their non-injected contralateral eyes are statistically significant (paired *t*-tests).

### The Kinetics of Dendritic Retraction of RGCs in NMDA Excitotoxicity

**FIGURE 6:**
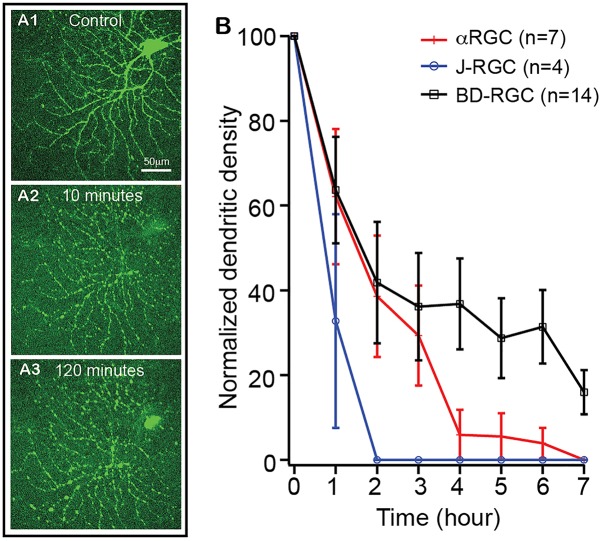
The kinetics of dendritic retraction of RGCs induced by NMDA excitotoxicity. The kinetics of dendritic retraction of αRGCs in Thy1-YFP retinas, BD-RGCs in BD:YFP retinas, and J-RGCs in JamB:YFP retinas were quantified using time lapse imaging on an *ex vivo* retinal preparation. **(A)** Images of an αRGC of a Thy1-YFP retina before **(A1)**, 10 min **(A2)** and 120 min **(A3)** after 10 min of 200 nmol/L NMDA perfusion. **(B)** Average dendritic density of αRGCs, BD-RGCs and J-RGCs as a function of time after bath application of 10 min of 200 nmol/L NMDA. Dendritic density was measured using a Sholl analysis (Xu et al., 2010) and was normalized to pre-NMDA application (0 h). The numbers of “*n*” indicate the number of cells analyzed for each type of RGCs.

It has been postulated that RGCs lose synaptic connections and dendritic processes before death in glaucoma and ONC models (Weber et al., 1998; Shou et al., 2003; Kuehn et al., 2005; Morgan et al., 2006; Leung et al., 2008; Weber et al., 2008; Liu et al., 2011; Della Santina et al., 2013; Feng et al., 2013; Ou et al., 2016). To test whether the extent of RGC dendritic retraction is correlated to the death rate of RGCs in a type-specific manner, we examined the kinetics of dendritic retraction of αRGCs, BD-RGCs and J-RGCs in response to NMDA excitotoxicity using time lapse imaging on an *ex vivo* retinal preparation (Xu et al., 2010). αRGCs can be easily recognized based on their dendritic pattern in Thy1-YFP mice (Xu et al., 2010). BD-RGCs and J-RGCs are recognized in BD:YFP and JamB:YFP retinas based on their YFP signaling and dendritic pattern (Figure 6A). Our results showed that J-RGCs lost 67% of their dendrites 1 h after 200 nmol/L NMDA application and completely lost all dendrites 2 h after NMDA application for 10 min (Figure 6B). The αRGCs, on the other hand, only lost 38% of their dendrites 1 h after NMDA application for 10 min but still lost all dendrites 7 h after NMDA application. Most noticeably, BD-RGCs lost 36% of their dendrites 1 h after NMDA application for 10 min but still maintained 18% of their dendrites 7 h after NMDA application (Figure 6B). Taken together with the results shown in Figure 5, these results demonstrate that the kinetics of dendritic retraction of RGCs does not correlate to the susceptibility of RGC death due to NMDA excitotoxicity.

## Discussion

Our results show that the susceptibility of different types of genetically identified RGCs to NMDA excitotoxicity varies significantly. The αRGCs are the most resistant RGCs to NMDA excitotoxicity while the J-RGCs are the most sensitive RGCs to NMDA excitotoxicity. These results strongly suggest that the differences in the genetic background of RGC types might provide valuable insights for the understanding of the selective vulnerability of RGCs to pathological insults and could assist in the development of strategies for protecting RGCs under disease conditions. In addition, the sequence of the morphological and molecular events during RGC death suggests that the initial insult of NMDA excitotoxicity might set off a cascade of events that is subsequently independent of the primary insults.

### Classification of RGC Types

Morphologically, RGCs are classified into about 20 types (Badea and Nathans, 2004; Völgyi et al., 2005; Kim et al., 2008; Kay et al., 2011), which is closely correlated to the function of RGCs (Cleland et al., 1975). For instance, based on RGC dendritic ramification in the IPL, RGCs are functionally divided into ON, OFF and ON-OFF types. Recent advances in optical imaging methods have provided an efficient way to record RGC light responses for functional classification of RGCs (Briggman and Euler, 2011; Briggman et al., 2011; Baden et al., 2016). More recently, RGCs have been classified into at least 40 types by combining morphological, functional and genetic features (Sanes and Masland, 2015; Rheaume et al., 2018).

This study includes four groups of RGCs with unique structural, functional and genetic features. The BD-RGCs are a type of ON-OFF direction-selective RGCs (DS-RGCs). In mouse retinas, there are four types of ON-OFF DS-RGCs, tuned to motion in ventral, dorsal, nasal, and temporal. BD-RGCs are sensitive to ventral motion (Kim et al., 2010; Trenholm et al., 2011). W3-RGCs are the smallest in size and the most numerous RGCs (Kim et al., 2010). There are at least two types of W3-RGCs: W3B, which are motion sensitive, and W3D, which remain physiologically uncharacterized (Zhang et al., 2012; Kim and Kerschensteiner, 2017). There are at least three types of αRGCs in mouse retinas (Pang et al., 2003; Estevez et al., 2012). Kcng4^Cre^:YFP mice express YFP in all three types of αRGCs, and some subsets of bipolar cells (Duan et al., 2015). There are three types of JamB expressing RGCs in mouse retina, which differ in dendritic tree morphology (Kim et al., 2008, 2010; Sanes and Masland, 2015). The JamB:YFP mice express YFP in two types of JamB expressing RGCs (J-RGCs). One type of J-RGCs orients its dendrites toward ventrally to form a polarized DF and is sensitive to directional movement, color-opponent responses, and orientation selective response (Kim et al., 2008, 2010; Joesch and Meister, 2016; Nath and Schwartz, 2017). The second type of J-RGCs has a symmetric DF and the function of them is not well characterized (Kim et al., 2008). In addition, YFP is expressed in about 12 morphological types of RGCs in the Thy1-YFP mice (Xu et al., 2010). Altogether, these transgenic mice provide a total of 8 RGC types individually or in small groups, including 1 DS-RGCs, 2 W3-RGCs, 3 αRGCs, 2 J-RGCs, and a mouse strain for a group of 12 types of RGCs.

### NMDA Excitotoxicity in Retinal Diseases

Glutamate excitotoxicity is thought to play a critical role in RGC death in many retinal diseases, such as glaucoma, diabetic retinopathy, optic nerve injury and retinal ischemia (Kuehn et al., 2005; Kwon et al., 2009; Tezel, 2013). Glaucoma is a chronic optic neuropathy characterized by progressive RGC axon degeneration and cell death. One proposed mechanism for glaucomatous damage describes increased pressure in the eye leading to glutamate-induced excitotoxicity. Consistently, elevated IOP increases the expression of NMDARs in DBA/2J mice (Dong et al., 2013) and the numbers of NMDAR positive RGCs are reduced parallel to the loss of RGC in a chronic elevated IOP model (Luo et al., 2009). In addition, a NMDA antagonist, memantine, significantly reduces RGC loss and the expression of NMDARs (WoldeMussie et al., 2002; Sánchez-López et al., 2018), suggesting that NMDARs are involved in the RGC death in glaucoma. Furthermore, elevated IOP activates NMDARs, which triggers mitochondria-mediated apoptosis through releasing of optic atrophy 1 (OPA1) (Ju et al., 2008). Blockade of glutamate receptor inhibits OPA1 release, increases Bcl-2 expression, decreases Bax expression, and block apoptosis in glaucomatous mouse retina (Ju et al., 2009).

There is an emerging body of evidence that suggests neurodegeneration is a key initial process in the development of diabetic retinopathy (DR) and RGC injury occurs prior to microvascular damage via multiple potential mechanisms including overstimulation of the NMDAR (Smith, 2002; Barber, 2003; Araszkiewicz and Zozulinska-Ziolkiewicz, 2016). For instance, there is an elevated aqueous/vitreous glutamate level in DR animal models and DR patients (Ambati et al., 1997; Kowluru et al., 2001). The immunoreactivities of NR1 and GluR2/3 are upregulated in RGCs of both patients with diabetes and experimental DR animals (Ng et al., 2004; Santiago et al., 2008). In addition, blocking of NMDAR protects RGCs against neurodegeneration in DR rats (Kusari et al., 2007).

In ONC models, the NMDA antagonists, memantine and MK-801, protect RGCs from death (Yoles et al., 1997; WoldeMussie et al., 2002). In addition, the AMPA-KA antagonist, DNQX, also protects RGCs after ONC (Schuettauf et al., 2000). Furthermore, MK-801 and other NMDAR antagonists also prevent RGC death by retinal ischemia, reduces the expression of the pro-degeneration gene Bad and significantly increases the pro-survival activity of the PI3K/Akt pathway in the retina (Russo et al., 2008). Therefore, NMDA excitotoxicity seems to participate in RGC death induced by both optic nerve injury and retinal ischemia.

### RGC Type-Specific Susceptibility to Retinal Diseases

The type-specific susceptibility of RGCs has been proposed as a factor in several retinal diseases. It has been proposed that the susceptibility of RGCs to glutamate excitotoxicity depends on soma size and retinal eccentricity. Larger RGCs at peripheral retina are more sensitive to kainate excitotoxicity while smaller RGCs at central retina are more sensitive to NMDA excitotoxicity (Vorwerk et al., 1999). In addition, intrinsically photosensitive melanopsin-expressing RGCs (ipRGCs) are also resistant to NMDA excitotoxicity (DeParis et al., 2012; Wang et al., 2018). In animal models of glaucoma, RGCs with large somata or big axon are more vulnerable to elevated IOP (Quigley et al., 1988; Glovinsky et al., 1991). Functionally, OFF RGCs appear to be more vulnerable to elevated IOP by reducing the strength of light responses and decreasing the size of the OFF receptive field (Della Santina and Ou, 2017; Sabharwal et al., 2017). OFF RGCs also exhibited higher rates of cell death and a more rapid decline in both structural and functional organizations compared to ON RGCs (Della Santina et al., 2013; El-Danaf and Huberman, 2015; Ou et al., 2016), but ON RGCs were more susceptible to elevated IOP than ON-OFF RGCs (Feng et al., 2013). In addition, the transient OFF αRGCs exhibited higher rate of cell death, while neither sustained OFF αRGCs nor sustained ON αRGCs have reduced synaptic activity due to elevated IOP (Ou et al., 2016). Similar to models with elevated IOP, OFF RGCs were more susceptible than ON RGCs to ONC, and ON sustained RGCs seem to be more susceptible than ON transient RGCs to ONC (Puyang et al., 2017). Among αRGCs, ipRGCs, DSRGCs and W3-RGCs, αRGCs seem to be the least susceptible type to ONC (Duan et al., 2015). These results are consistent with our observation in this study and support the notion that glutamate excitotoxicity could play critical roles in RGC death of optic nerve injury.

Inconsistent with some previous reports, our results do not provide a clear correlation between RGC morphology and susceptibility to NMDA excitotoxicity. Among the 4 groups of genetically identified RGCs tested in this study, their susceptibility to NMDA excitotoxicity seems not directly correlate to the size of their soma and DF. This is evident that both BD-RGCs and J-RGCs have much higher susceptibility to NMDA excitotoxicity than αRGCs, which are known to have the biggest size of soma and DF, and W3-RGCs, which are the RGCs with the smallest size of soma and DF (Kim et al., 2010). This is opposite to the observations by several previous studies (Quigley et al., 1988; Glovinsky et al., 1991; Vorwerk et al., 1999). It was also reported that OFF RGCs appear to be more vulnerable to elevated IOP and ONC than ON RGCs (Della Santina et al., 2013; El-Danaf and Huberman, 2015; Ou et al., 2016; Della Santina and Ou, 2017; Puyang et al., 2017; Sabharwal et al., 2017), while ON RGCs are more susceptible to elevated IOP than ON-OFF RGCs (Feng et al., 2013). However, the ON and OFF inputs seem not play a critical role in NMDA-induced RGC death to BD-RGCs, which are ON-OFF RGCs (Kim et al., 2010; Trenholm et al., 2011), and J-RGCs, which are OFF-RGCs (Kim et al., 2008, 2010; Joesch and Meister, 2016; Nath and Schwartz, 2017).

An important question is what the underlying reasons contribute to these inconsistent observations. At least two important factors might play significant roles to this RGC type-specific susceptibility: the way how the RGC types are determined and the types of pathological insults. RGCs have been classified into types based on morphological, functional and genetic properties (Cleland et al., 1975; Badea and Nathans, 2004; Völgyi et al., 2005; Kim et al., 2008; Briggman and Euler, 2011; Briggman et al., 2011; Kay et al., 2011; Sanes and Masland, 2015; Baden et al., 2016; Rheaume et al., 2018). Most previous studies of RGC type-specific susceptibility are based on morphological and functional classification (Quigley et al., 1988; Glovinsky et al., 1991; Vorwerk et al., 1999; Della Santina et al., 2013; Feng et al., 2013; El-Danaf and Huberman, 2015; Ou et al., 2016; Della Santina and Ou, 2017; Puyang et al., 2017; Sabharwal et al., 2017). Because these morphologically and functionally classified RGC types are likely to have heterogeneous gene expression profiles and, if the gene expression profiles of RGCs contribute to the type-specific susceptibility, how the RGCs are grouped into types could have significant influence on the observed susceptibility.

Although very few studies have directly compared the susceptibility of the same type of RGCs to different pathological insults, it is plausible to assume that the underlying molecular mechanisms of RGC death induced by different pathological insults might be different due to the nature of insults, such as NMDA excitotoxicity elevates intracellular calcium while ONC reduces axonal transportation. Therefore, the susceptibility of the same type of RGCs might vary significantly to different types of pathological insults. Consistent with this idea, our unpublished data of an ongoing study demonstrate that the susceptibility of the four types RGCs tested in this study varies dramatically with types of injuries. In responding to ONC, BD-RGCs have the lowest susceptibility while W3-RGCs have the highest susceptibility, which is opposite to the ranking of susceptibility to NMDA excitotoxicity. Therefore, we propose that the susceptibility of different types of RGCs is likely to be determined by an interaction between the pathological insults and cell intrinsic response mechanisms. Different types of injuries might trigger different intrinsic response mechanisms in different types of RGCs, which might have different efficacy in activation of the cell death processes in different types of RGCs. If this is a general rule for type-specific RGC death in retinal diseases, it may not be reliable to predict the pattern of RGC death in one disease based on patterns of other diseases.

### The Sequence of RGC Degeneration

Retinal ganglion cells lose synapses prior to a reduction in synaptic activity, leading to dendritic shrinkage and eventually cell death in glaucoma models of primates, cats, rats and human patients and animal models of ONC (Weber et al., 1998; Shou et al., 2003; Kuehn et al., 2005; Morgan et al., 2006; Leung et al., 2008; Weber et al., 2008; Liu et al., 2011; Della Santina et al., 2013; Feng et al., 2013; Ou et al., 2016). Carefully studying the sequence of structural, functional and molecular changes of injured RGCs could provide critical insights into the underlying mechanisms of RGC death and shed light on potential treatments aimed at reversing or slowing RGC degeneration. The present study demonstrates that many RGCs that lost all of their dendrites remained CASP3-negative while hardly any RGCs expressed CASP3 before completely losing their dendrites. The sequence of these morphological and molecular events suggests that the initial insult of NMDA excitotoxicity might only induce the loss of RGC dendrites through altering of the synaptic activity. Because this sequence of events seems to be consistent across several different experimental disease models, the initial insult in retinal diseases may set off a cascade of events that is subsequently independent of the primary insults. Regardless the underlying mechanisms, the kinetics of dendritic retraction of RGCs does not directly correlate to the susceptibility of type-specific RGC death. Because RGCs lose dendrites prior to cell death, using the dendritic morphology, the ramification patterns of RGC dendrites or patterns of light responses of RGCs to identify types of the cells under disease conditions might be misleading. The use of genetic markers provides a more reliable approach to identify the types of RGC under disease conditions. Therefore, our results provide valuable insights into the type-specific susceptibility of RGCs to NMDA excitotoxicity.

## Data Availability

All datasets generated for this study are included in the manuscript and/or the supplementary files.

## Author Contributions

IC and NY collected and analyzed the data and prepared the manuscript. BL and KH collected and analyzed the data. PW contributed the animal preparation and resource management. NT designed the experiments, analyzed the data, prepared the manuscript, and managed the research fund.

## Conflict of Interest Statement

The authors declare that the research was conducted in the absence of any commercial or financial relationships that could be construed as a potential conflict of interest.
